# A case report: long-term survival cases of nasopharyngeal carcinoma with breast metastasis

**DOI:** 10.3389/fonc.2026.1801777

**Published:** 2026-04-22

**Authors:** Xiaojuan Lu, Xingxing Lv, Junyan Wan, Juan Chen, Hong Lu

**Affiliations:** 1 Department of Oncology And Hematology, People’s Hospital of Leshan, Leshan, China; 2 Urinary Surgery, People’s Hospital of Leshan, Leshan, China

**Keywords:** breast metastasis, case report, Epstein-Barr virus (EBV) infection, nasopharyngeal carcinoma, secondary breast tumor

## Abstract

**Background:**

Metastatic malignancies in the breast most commonly originate from cancers of adjacent organs, including lymphoma, melanoma, and lung cancers, while metastases from distant primary sites, including gastrointestinal, head and neck, and pelvic malignancies, are less likely to metastasize to the breast. Nasopharyngeal carcinoma (NPC) with breast metastases is rare. This report describes a patient with pathologically confirmed NPC with breast metastasis who achieved favorable treatment outcomes.

**Case presentation:**

A 50-year-old Chinese man with a history of NPC developed multiple systemic metastases. Histopathological examination of a breast lesion confirmed NPC metastasis. After systemic chemotherapy combined with immunotherapy, the patient achieved disease remission and was alive at the time of reporting.

**Conclusion:**

This case also serves as a reminder of the importance of re-biopsy for recurrent or metastatic lesions. These observations emphasize the need for further research regarding uncommon metastatic patterns of NPC to refine treatment strategies and improve clinical outcomes.

## 1 Introduction

Invasive carcinomas account for over 95% of all malignant breast tumors. Besides primary breast cancer, malignant tumors of the breast are caused by metastasis from the contralateral breast. Hematologic malignancies, such as lymphoma, and extramammary tumors, such as melanoma have also been reported to metastasize to the breast (1). However, metastases represents less than 2% of all breast malignancies, and cases originating from nasopharyngeal carcinoma (NPC) are particularly rare (2). NPC is an epithelial malignant tumor that originates from the mucosa of the nasopharynx. It mainly occurs on the superior and lateral walls of the nasopharynx, especially in the pharyngeal recess. The causes of the disease include genetic and environmental factors, with Epstein-Barr virus (EBV) infection being especially prevalent. Unhealthy lifestyle habits, such as smoking and consumption of pickled foods, may also contribute to nasopharyngeal cancer (3). NPC exhibits a high propensity for locoregional invasion and distant metastasis, with the most common metastatic sites being the bones, lungs, and liver (4). In this case report, we describe a rare case of pathologically confirmed breast metastasis of NPC with a long-term survival of nearly 7 years following initial diagnosis.

## 2 Case report

**Figure 1:**
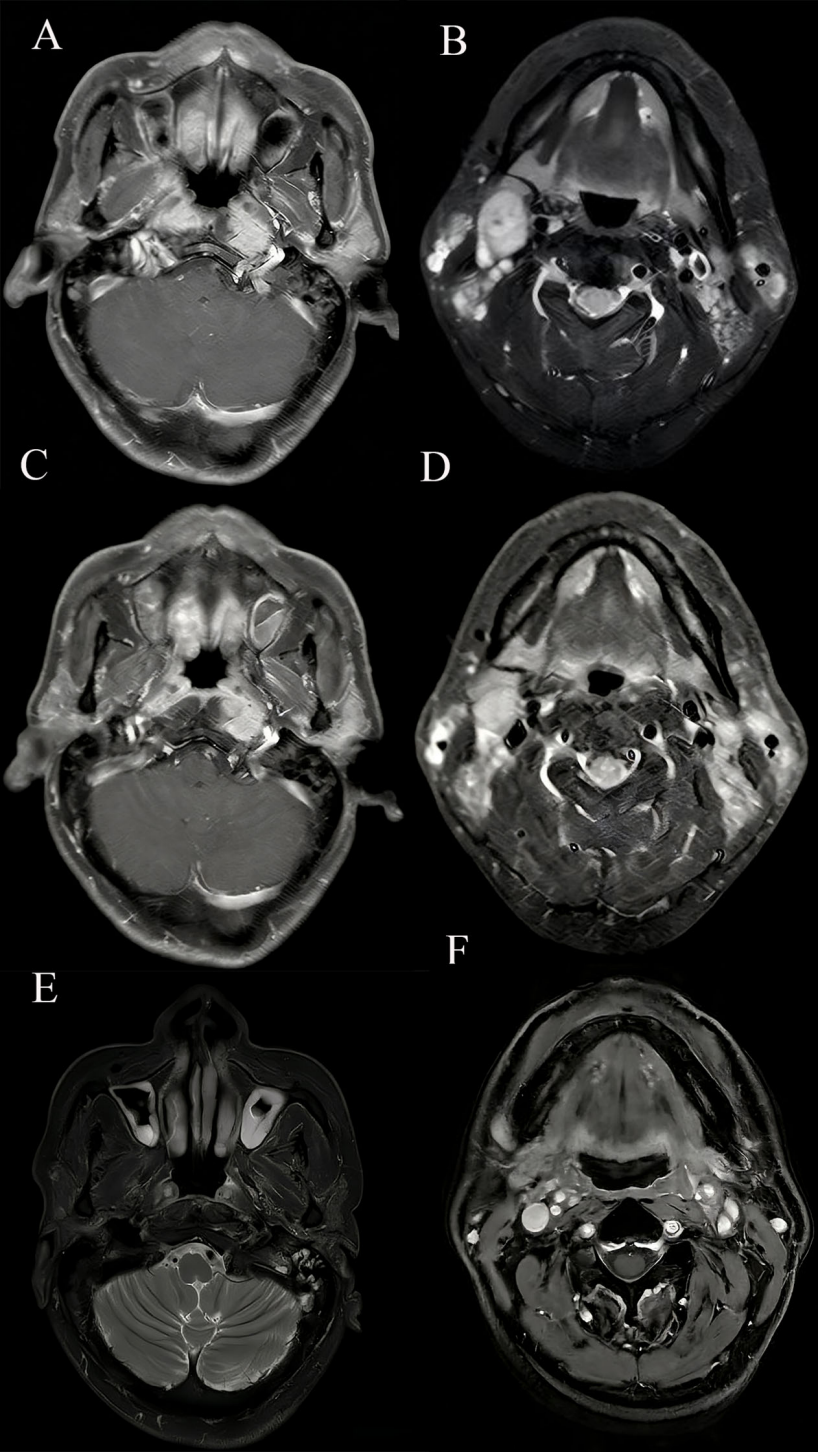
Computed tomography (CT) images obtained during the course of treatment. **(A, B)** CT images of the lesion at initial presentation: nasopharyngeal lesion **(A)** and lymph node **(B)**. **(C, D)** CT images after three cycles of chemotherapy. **(E, F)** Current CT findings on January 16, 2026, showing no evidence of tumor recurrence.

Two years prior to his diagnosis, a 50-year-old man discovered a painless 2×3 cm mass in his right neck without any obvious cause, which gradually resolved after self-administration of medication (details unknown). One year later, the patient had occasional headaches but did not seek medical attention. Later, he developed recurrent blood-tinged nasal discharge, ongoing right facial numbness, diplopia, and impaired taste, which led to a nasopharyngeal biopsy. Biopsy revealed non-keratinizing undifferentiated carcinoma (WHO type III) with positive EBV-encoded DNA *in situ* hybridization. Immunohistochemical staining results were as follows: P63(+), CK5/6(+), EGFR (+), EBV (+), P16 (+), and Ki67 (+; approximately 60%). Contrast-enhanced magnetic resonance imaging (MRI) of the nasopharynx and neck demonstrated thickening of the mucosa at the posterior aspect of the superior nasopharynx (approximately 8.8 mm) (**Figure 1A**) and involvement of the bilateral parapharyngeal spaces and the longus capitis muscle, along with lymph node metastasis on both sides of the neck (largest measuring 2.4×1.6 cm in level II on the right side) (**Figure 1B**). Further imaging studies were performed to evaluate for possible metastasis in other parts of the body.

One month after his diagnosis, the patient underwent four cycles of induction TPF chemotherapy (docetaxel 60 mg/m^2^ intravenously on day 1, cisplatin 60 mg/m^2^ intravenously on day 1, and fluorouracil 600 mg/m^2^ intravenously on days 1–5). After the third cycle, imaging showed a reduction in size of both the nasopharyngeal (**Figure 1C**) and cervical lymph node lesions (**Figure 1D**). Three months later, intensity-modulated radiation therapy was administered to the nasopharynx and regional lymph nodes (data not shown). Two additional cycles of TPF chemotherapy were completed, after which the patient’s EBV DNA levels were negative. Follow-up evaluation showed no evidence of recurrence or metastasis.

**Figure 2:**
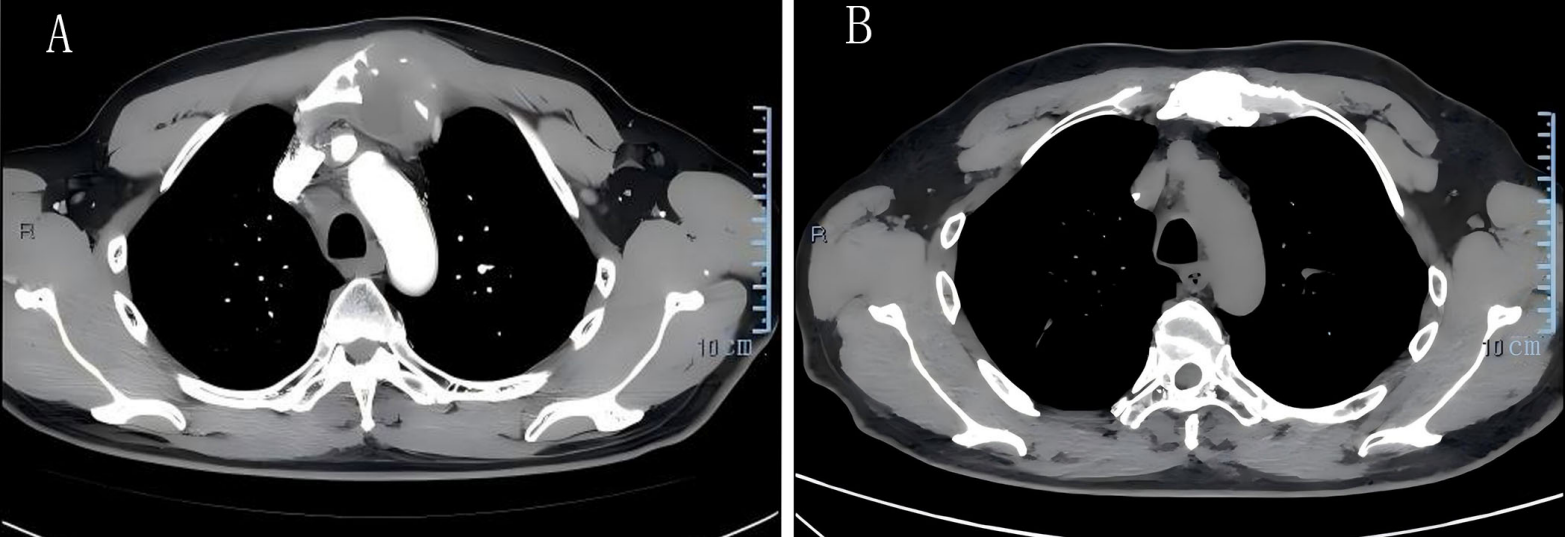
Computed tomography (CT) images of the sternal metastasis. **(A)** Initial lesion at the manubrium of the sternum. **(B)** Significant shrinkage of the lesion on January 16, 2026.

**Figure 3:**
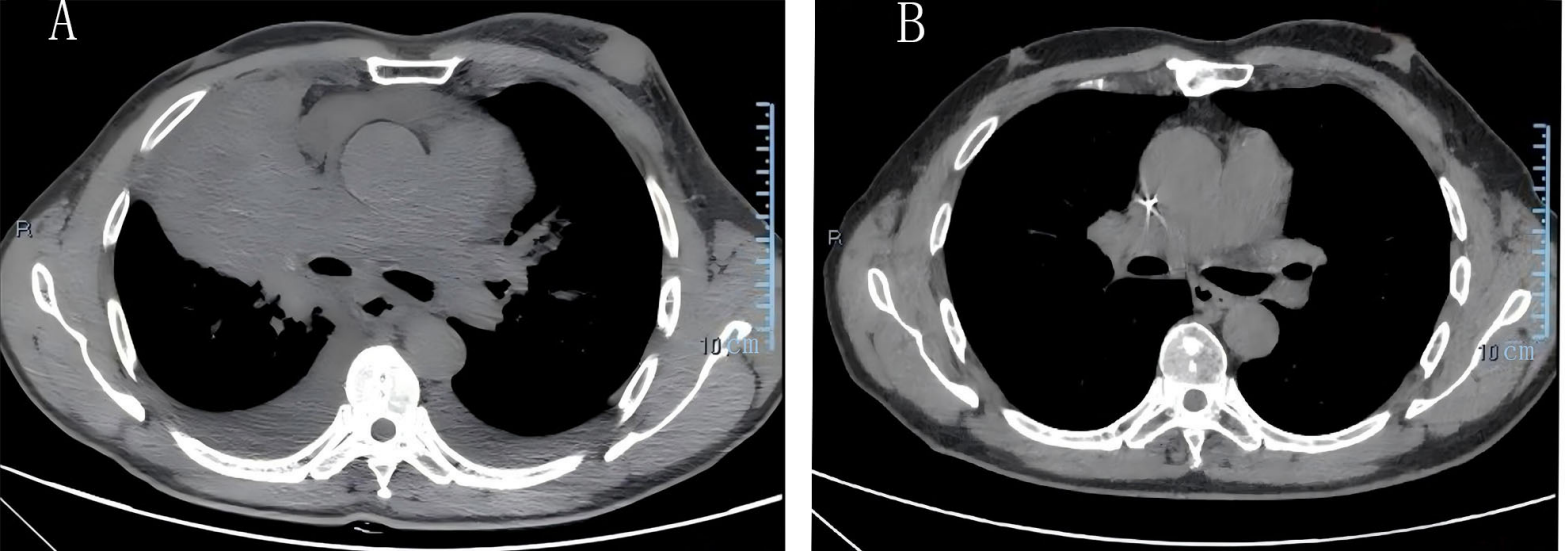
Computed tomography (CT) images of the breast lesion. **(A)** Initial detection of the breast lesion in August 2023. **(B)** The lesion had completely resolved by January 16, 2026.

**Figure 4:**
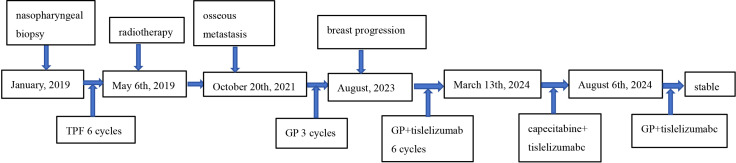
Timeline of the patient’s treatment.

Thirty months after his diagnosis of NPC, the patient developed a progressively enlarging, painful mass adjacent to his manubrium. Contrast-enhanced computed tomography (CT) scans revealed lytic bone destruction of the left manubrial aspect, with an associated soft-tissue mass measuring 4.9 cm × 4.3 cm proximal to major mediastinal vessels (**Figure 2A**), along with mediastinal lymphadenopathy. EBV DNA was positive. Bone scintigraphy demonstrated increased radiotracer uptake in the manubrium, consistent with osseous metastasis. CT-guided biopsy revealed inflammatory cell infiltration and atypical epithelial cells at the margins. Immunohistochemical results indicated CK (+), CK5/6 (+), P63 (+), EGFR (+), Ki67 (+, approximately 70%), LCA (−), and S-100 (−). The confirmation of metastatic NPC in the parasternal area indicated disease progression. The patient underwent three cycles of gemcitabine and cisplatin chemotherapy (gemcitabine 1000 mg/m^2^ intravenously on days 1 and 8 and cisplatin 75 mg/m^2^ intravenously on day 1), after which the EBV DNA became undetectable. However, the patient voluntarily discontinued further treatment. Two years after the metastasis was detected, the patient developed fatigue and a cough. A chest CT showed a consolidation in the upper and middle lobes of the right lung, suspicious parenchymal abnormalities in the lower lobe of the right lung and the upper lobe of the left lung, mediastinal and bilateral axillary lymphadenopathy, ongoing manubrial bone metastasis, and new metastatic involvement in the left breast. Pericardial and bilateral pleural effusions were also observed (**Figure 3A**). The EBV DNA test was positive. A breast biopsy confirmed non-keratinizing squamous cell carcinoma morphologically consistent with metastatic NPC, confirming disease progression. Immunohistochemistry revealed ER (−), PR (−), Calponin (−), P63 (+), CK5/6 (+), E-Ca (+), P120 membrane (+), Her-2 (0, negative), Ki-67 (+, approximately 50%), Pan-CK (+), EGFR (+), GATA-3 (+) and EBER1/2(+). The patient underwent two cycles of gemcitabine and cisplatin chemotherapy and immunotherapy with tislelizumab (tislelizumab 200 mg intravenously on day 1; gemcitabine 1000 mg/m^2^ intravenously on days 1 and 8, and 75 mg/m^2^ cisplatin intravenously on day 1). Posttreatment imaging revealed improved pulmonary consolidation, reduced lymphadenopathy, and decreased pleural effusion. However, a new metastatic lesion with a pathological fracture was identified in the sternum. EBV DNA levels normalized, and a partial response was achieved. The patient completed four additional cycles of gemcitabine and cisplatin chemotherapy combined with tislelizumab, followed by maintenance therapy with gemcitabine and tislelizumab. Subsequently, the patient refused intravenous chemotherapy. Therefore, after four cycles of treatment, capecitabine combined with tirapamil was used for continued maintenance therapy. The patient then developed immune-related pneumonitis, which improved following tislelizumab discontinuation and prednisone therapy initiation. Due to local insurance reimbursement requirements, the patient resumed low-dose gemcitabine and cisplatin chemotherapy with tislelizumab. At the most recent follow-up, the patient’s condition was stable. Post-radiotherapy changes of the nasopharynx and neck are shown in **Figures 1E, F**. The sternum and breast lesions were significantly reduced in size (**Figures 2B**, **3B**). No signs of distant metastasis were observed. However, the EBV DNA is currently positive. The patient’s treatment timeline is shown in **Figure 4**.

## 3 Discussion

Based on prior reports, the incidence of metastasis of non-breast malignancies to the breast is <2% (5). Metastases originating from head and neck cancers are particularly uncommon, and NPC-related breast metastasis is a rare clinical entity. Since the first case was reported in 1991 (2), approximately 14 cases have been documented in the literature to date (6). A retrospective study conducted in southern China reported that NPC accounted for 18.2% (4/22) of breast metastases from extramammary solid tumors, likely reflecting the high endemic prevalence of NPC in this region (7). The onset age is typically between 50 and 60 years, with an average age of 43 years (8). Although most of the reported cases are female, male cases with breast metastases have been documented (9), and the prognosis for these cases is even worse.

Typical clinical features of breast metastases from NPC include solitary, firm, painless, and mobile masses in the breast (68.2%), sometimes accompanied by ipsilateral axillary lymphadenopathy. Multiple rubbery subcutaneous nodules may be present. Typically, breast metastasis occurs 6 to 56 months after the diagnosis of NPC, with a median latency period of 27 months. Nearly 50% of the patients develop breast metastasis within 2 years after the diagnosis. Most metastases are unilateral, with similar frequencies observed in the left and right breasts (45.5% and 36.4%, respectively). The breast may represent the sole site of metastatic disease (27.3%) or breast metastasis may occur as part of disseminated metastases (31.8% with the involvement of other sites).

Histologically, breast metastasis from NPC typically manifests as poorly differentiated squamous cell carcinoma, usually lacking keratinization. The cancer cells usually form nests of varying sizes and irregular shapes, with no obvious layering. The cancer cells appear polygonal or oval, with abundant cytoplasm and clear boundaries. A few cancer cells may also show intercellular bridges (9). The diagnosis of NPC mainly relies on immunohistochemical findings. The typical immunoprofile includes positive pan-cytokeratin expression confirming epithelial origin; negative CK7 (to exclude CK7-positive carcinomas such as breast cancer) (10); negative CK20 (to rule out gastrointestinal origin); negative estrogen receptor and progesterone receptor (to exclude hormone receptor-positive breast cancer); negative HER2 (to exclude HER2-overexpressing breast cancer) (11); and negative S-100, HMB-45, leukocyte common antigen, and thyroid transcription factor-1 (to exclude melanoma, lymphoma, and lung adenocarcinoma, respectively). The 2023 breast lesion biopsy demonstrated squamous cell carcinoma morphology on immunohistochemistry, with positive expression of EBER and p63, findings that more strongly support metastasis from nasopharyngeal carcinoma to the breast. In some cases, neuroendocrine markers, such as chromogranin A, synaptophysin, and CD56, may be expressed. Mammography typically reveals spherical masses with indistinct or spiculated margins that mimic primary breast cancer. Ultrasonography may reveal oval or lobulated hypoechoic lesions that lack the classic imaging characteristics of malignancy. Contrast-enhanced CT or MRI shows heterogeneously enhancing masses, occasionally with calcifications (12). Ultrasound and nuclear medicine imaging often show irregularly shaped nodules, and the BI-RADS classification is often 4b-5, indicating a high suspicion of malignancy. Distinguishing metastatic breast involvement from primary breast cancer remains challenging due to the overlapping clinical and radiological presentations. The differential diagnoses include primary breast carcinoma (the most common misdiagnosis), metastatic melanoma, lymphoma, and sarcoma. A definitive diagnosis relies on histopathological evaluation and comprehensive immunohistochemical profiling, with tissue acquisition via core needle biopsy. The correlation between a patient’s known history of NPC and extensive immunohistochemical marker analysis is essential for accurate diagnosis. Throughout the diagnosis, treatment, and follow-up of NPC, serial monitoring of serum EBV DNA levels is crucial for assessing disease progression and treatment response. In this patient, EBV DNA was undetectable during periods of effective treatment. However, when serum EBV DNA became positive later, imaging results indicated that the disease had progressed, consistent with clinical expectations. This may be related to the persistent EBV infection altering the tumor microenvironment, thereby promoting cancer cell proliferation and metastasis.

At present, there is no definite treatment plan for breast metastasis caused by nasopharyngeal cancer, and therapeutic decisions should be made through multidisciplinary team discussions. The primary treatment strategy includes platinum-based systemic chemotherapy, which is the primary palliative approach. PD-1 inhibitors combined with gemcitabine and cisplatin have become the first-line standard of care for patients with recurrent or metastatic NPC. Targeted therapies directed against specific molecular markers are currently being investigated (13). Palliative radiotherapy (such as 40 Gy in 20 fractions) may be considered for breast metastases; however, the role of surgical resection remains controversial. Although one study suggested a trend toward improved overall survival after surgery, further validation is required. Due to the rarity and poor prognosis of this condition, individualized treatment planning through multidisciplinary collaboration is strongly recommended. The primary treatment goal is palliative care, aimed at controlling symptoms and prolonging survival. Under current treatment methods, the therapeutic effect is unsatisfactory. The median overall survival period is around 14 months (range, 2–74 months), and most previous studies report survival of less than 3 years (6). This patient has achieved a survival duration of up to 7 years post-treatment, with effective lesion control and manageable treatment-related toxicities and adverse events. The leading causes of death are systemic deterioration and respiratory failure. Poor prognostic factors include extensive metastatic burden, male sex (although data are limited and trends remain unclear), and poor treatment compliance. In the present case, the patient developed bone and lymph node metastases 2 years after his diagnosis of NPC and was found to have multiple breast and lung metastases 4 years after the initial diagnosis. Currently, the disease is under stable control following systemic chemotherapy combined with immunotherapy. After treatment, EBV DNA levels rapidly became undetectable, indicating high treatment sensitivity. This patient was diagnosed with breast metastasis from NPC in September 2023. Following treatment with chemotherapy combined with immunotherapy, the lesion was successfully controlled, and the patient achieved complete remission. The progression-free survival with this combined therapy has reached 28 months. Overall, from the time of initial diagnosis to the present, the patient has experienced a continuous survival benefit of 84 months, representing the longest survival and the most significant therapeutic benefit reported to date. This outcome also highlights the strong heterogeneity of NPC. The main adverse events observed during treatment included immune-related pneumonia, hypothyroidism, leukopenia, neutropenia, nausea, and vomiting, all graded as 1–2 adverse events. After symptomatic treatment, the overall safety profile of the treatment was acceptable.

## 4 Conclusion

Breast metastasis from NPC represents an extremely rare clinical entity with significant diagnostic challenges. Clinical and imaging features closely resemble those of primary breast cancer, necessitating pathological examination and immunohistochemical analysis for a definitive diagnosis. Due to limited experience in the diagnosis and management of such cases, there is currently no definitive consensus on the optimal treatment approach. Individualized palliative strategies developed through multidisciplinary collaboration remain the cornerstone of care. For patients with NPC who develop breast lumps during the course of their disease, clinicians must maintain a high index of suspicion for metastatic disease and promptly perform a puncture biopsy to confirm the pathology. Inappropriate treatments due to misdiagnosing these lesions as primary breast tumor should be avoided. When the patient’s condition allows, combined treatment modalities may offer an effective therapeutic strategy.

## Data Availability

The original contributions presented in the study are included in the article/supplementary material. Further inquiries can be directed to the corresponding author.
